# Anandamide Attenuates Th-17 Cell-Mediated Delayed-Type Hypersensitivity Response by Triggering IL-10 Production and Consequent microRNA Induction

**DOI:** 10.1371/journal.pone.0093954

**Published:** 2014-04-03

**Authors:** Austin R. Jackson, Prakash Nagarkatti, Mitzi Nagarkatti

**Affiliations:** Department of Pathology, Microbiology, and Immunology, University of South Carolina School of Medicine, Columbia, South Carolina, United States of America; University Hospital Würzburg, Germany

## Abstract

Endogenous cannabinoids [endocannabinoids] are lipid signaling molecules that have been shown to modulate immune functions. However, their role in the regulation of Th17 cells has not been studied previously. In the current study, we used methylated Bovine Serum Albumin [mBSA]-induced delayed type hypersensitivity [DTH] response in C57BL/6 mice, mediated by Th17 cells, as a model to test the anti-inflammatory effects of endocannabinoids. Administration of anandamide [AEA], a member of the endocannabinoid family, into mice resulted in significant mitigation of mBSA-induced inflammation, including foot pad swelling, cell infiltration, and cell proliferation in the draining lymph nodes [LN]. AEA treatment significantly reduced IL-17 and IFN-γ production, as well as decreased RORγt expression while causing significant induction of IL-10 in the draining LNs. IL-10 was critical for the AEA-induced mitigation of DTH response inasmuch as neutralization of IL-10 reversed the effects of AEA. We next analyzed miRNA from the LN cells and found that 100 out of 609 miRNA species were differentially regulated in AEA-treated mice when compared to controls. Several of these miRNAs targeted proinflammatory mediators. Interestingly, many of these miRNA were also upregulated upon *in vitro* treatment of LN cells with IL-10. Together, the current study demonstrates that AEA may suppress Th-17 cell–mediated DTH response by inducing IL-10 which in turn triggers miRNA that target proinflammatory pathways.

## Introduction

Cannabinoids are compounds derived from the *Cannabis sativa* plant and exert many effects on the immune system [1], [2], [3]. Cannabinoids have potential as therapeutic agents in several different disease conditions, including experimental autoimmune hepatitis, Multiple Sclerosis, and Graft vs. Host Disease [1], [2]–[5]. In addition, there is an endogenous network of cannabimimetic lipids called the endogenous cannabinoid system that serve as signaling molecules regulating many processes in the Central Nervous System. The major members of this family of compounds include Arachidonyl Ethanolamide [AEA, Anandamide] and 2-Arachidonyl Glycerol [2-AG]. These compounds act by activating specific receptors called cannabinoid receptors, of which there are two, CB1 and CB2. The CB receptors are members of the G-Protein coupled receptor family and mediate their effects through a series of G proteins and adaptors [6]. While endocannabinoids are most concentrated in the central nervous system, they also mediate peripheral effects. Among these, changes in the endocannabinoid system have been reported in liver injury [7], colitis [8], and diabetes [9]. When both cannabinoid receptors were knocked out, mice were more prone to developing allergic hypersensitivity [10]. This report suggested a role of the endogenous cannabinoid system in regulation of allergic inflammation.

Methylated Bovine Serum Albumin [mBSA] induces a Type-IV delayed-type hypersensitivity [DTH] characterized predominantly by Th17-driven inflammation and swelling at the site of antigen rechallenge [11]. This model of hypersensitivity has been used as a way to study the development of Th17 cells *in vivo* by injecting both intra-articularly into mice to induce arthritis [12] as well as subcutaneously to sensitize, and rechallenge the animals in the footpads [13] or the ear to induce DTH[14]. Th17 cells, besides their role in microbial immunity, are directly involved in the pathogenesis of autoimmune diseases such as arthritis [15], Experimental Autoimmune Encephalomyelitis [EAE] [16], [17], and myocarditis [18]. Thus, Th17 cells are a target for pharmaceutical intervention because they promote a pro-inflammatory environment.

MicroRNAs are a class of small nucleic acids ranging 19–25 nucleotides long that have an important function regulating expression of genes [19]–[22]. These small RNA species regulate expression of genes by complementary base-pairing in the 3′UTR of expressed mRNA and alter the stability of the targeted mRNA [23], [24]. Many studies have shown the effect of miRNAs on T helper cell differentiation, including the effect of let-7e on Th1/Th17 differentiation in EAE [25], miRNA 155 on Th17 differentiation [26], and miR10a induced by TGF-β that inhibits the plasticity of T cell subsets [27]. miRNA regulation in airway inflammation and asthma has also been described [28]. Moreover, miRNA 21 has been shown to be important for chemical-induced hypersensitivity [29]. However, there are no studies showing how miRNA can regulate IL-17-mediated DTH reaction or endocannabinoid-mediated immunosuppression.

In this study, we show that treatment with AEA can attenuate mBSA-induced hypersensitivity through secretion of IL-10 involving miRNA that target IL-17 and other proinflammatory pathways. These studies not only help understand the mechanism of action of endocannabinoids but also shed light on potential therapeutic targets against inflammatory disorders.

## Materials and Methods

### Reagents

AEA was obtained from the National institute on Drug Abuse [NIDA], National Institutes of Health [Bethesda, MD]. Methylated BSA [mBSA] and Freund's Complete Adjuvant [CFA] was purchased from Sigma Aldrich. CD3-Phycoerthyrin-Cyanin 7[PE-Cy7], CD4-PE, and IL17A FITC, monoclonal antibody was purchased from Biolegend. Annexin V-PI staining kit was purchased from biolegend. Complete RPMI was made according to the following formulation: 1% Penicillin/Streptomycin, 10% Fetal bovine Serum, 20 mM Glutamine, 50μM β-Mercaptoethanol, and 10 mM HEPES.

### Mice

Female adult C57Bl/6 mice were purchased from National Cancer Institute, National Institute of Health [Frederick, MD] and bred under appropriate conditions. All protocols were approved by the University of South Carolina Institutional Animal Care and Use Committee [IACUC] and mice were housed under specific pathogen-free conditions.

### Induction of mBSA-Induced Type-IV hypersensitivity and AEA treatment

Groups of 5 Mice were sensitized to mBSA by giving a subcutaneous injection of 150 μg mBSA emulsified in CFA, as described previously [15]. Two injections were given to each mouse, one in each hind flank. After 6 days, mice were rechallenged in the right footpad with 200μg mBSA in PBS and in the left footpad with PBS only. Percent increase in swelling was determined by the following equation: [thickness of mBSA footpad - thickness of PBS footpad]/thickness of PBS footpad × 100%. Mice were treated with either 40 mg/kg body weight AEA or vehicle, as described before [1] for 2 days prior to rechallenge. Mice were sacrificed 24 hours after rechallenge.

### Th17 intracellular stain

Cells were isolated from the draining lymph nodes [LN] 24 hours after mBSA rechallenge. These cells were stained for IL-17A using Intracellular Cytokine Staining Kit [BD biosciences] per manufacturers specifications. Briefly, cells were first stained for CD4-PE and then fixed/permeablized. Next, the cells were stained for IL-17A-FITC and analyzed on an FC500 Cytometer.

### Histology

Footpads from AEA-treated and vehicle-treated mice were isolated for histological examination. The feet were cut distal to the ankle and decalcified/fixed in Cal-Rite [Fisher scientific]. The slides were stained with H&E for generic architecture and immune cell infiltration.

### In Vitro mBSA restimulation

Mice were sensitized to mBSA as described above, but sacrificed on day 6. Cells were isolated from LN, plated at 1×10^6^ cells/well, cultured in the presence of 40 μg/ml mBSA, and treated with either vehicle or AEA.

### Cytokine Analysis

Cells from the LN of mBSA-sensitized mice were prepared as before and cultured overnight without mBSA to analyze for spontaneous cytokine secretion. Next, culture supernatants were analyzed for pro-inflammatory cytokines IL-17, IFN-γ, and IL-10 using ELISA kit per manufacturer's specifications [Biolegend].

### miRNA array and analysis

miRNA analysis was performed as described previously [27]. Total RNA was isolated from draining LNs from vehicle-treated and AEA-treated mice by using the miRNAeasy total RNA isolation kit and analyzed for purity using A260/280 readings. RNA purity for each sample was 1.7–1.9. Samples were analyzed using Affymetrix miRNA 1.0, allowing for analysis of 609 miRNA species. Target prediction was performed on microRNA.org MiRanda and MirSvR databases and pathway analysis was performed using Ingenuity Pathway Analysis [IPA] Software. miRNA differences were validated using QuantiTech Sybr Green PCR kit and miRNA primer assays [Qiagen].

### IL-10 neutralization

Mice were sensitized to mBSA and treated with vehicle or AEA as described above. One hour after each treatment with AEA or vehicle, mice were given 50 μg monoclonal antibodies to IL-10 [JES5-16E3] or isotype control. Mice were rechallenged and footpads were measured as before.

### In vitro IL-10 treatment

LN from mBSA-sensitized mice were prepared as before and plated in the presence of 40 μg/ml mBSA and increasing doses of IL-10 [10 or 20 ng/ml]. Cells were assayed for Th17 induction and miRNA profile as before.

### Real-Time PCR

RNA was isolated from draining LN using the miRNeasy RNA isolation kit [Qiagen]. cDNA synthesis was performed using iScript cDNA synthesis kit [Biorad]. All samples were run on a BioRad CXP96 Thermal Cycler.

### Statistical analysis

All statistical analyses were performed using Graphpad Software. Swelling data were analyzed using the Mann-Whitney U test, while all other data were analyzed by either the Student's t test or ANOVA, as appropriate.

## Results

### AEA mitigates mBSA-induced inflammation

**Figure 1:**
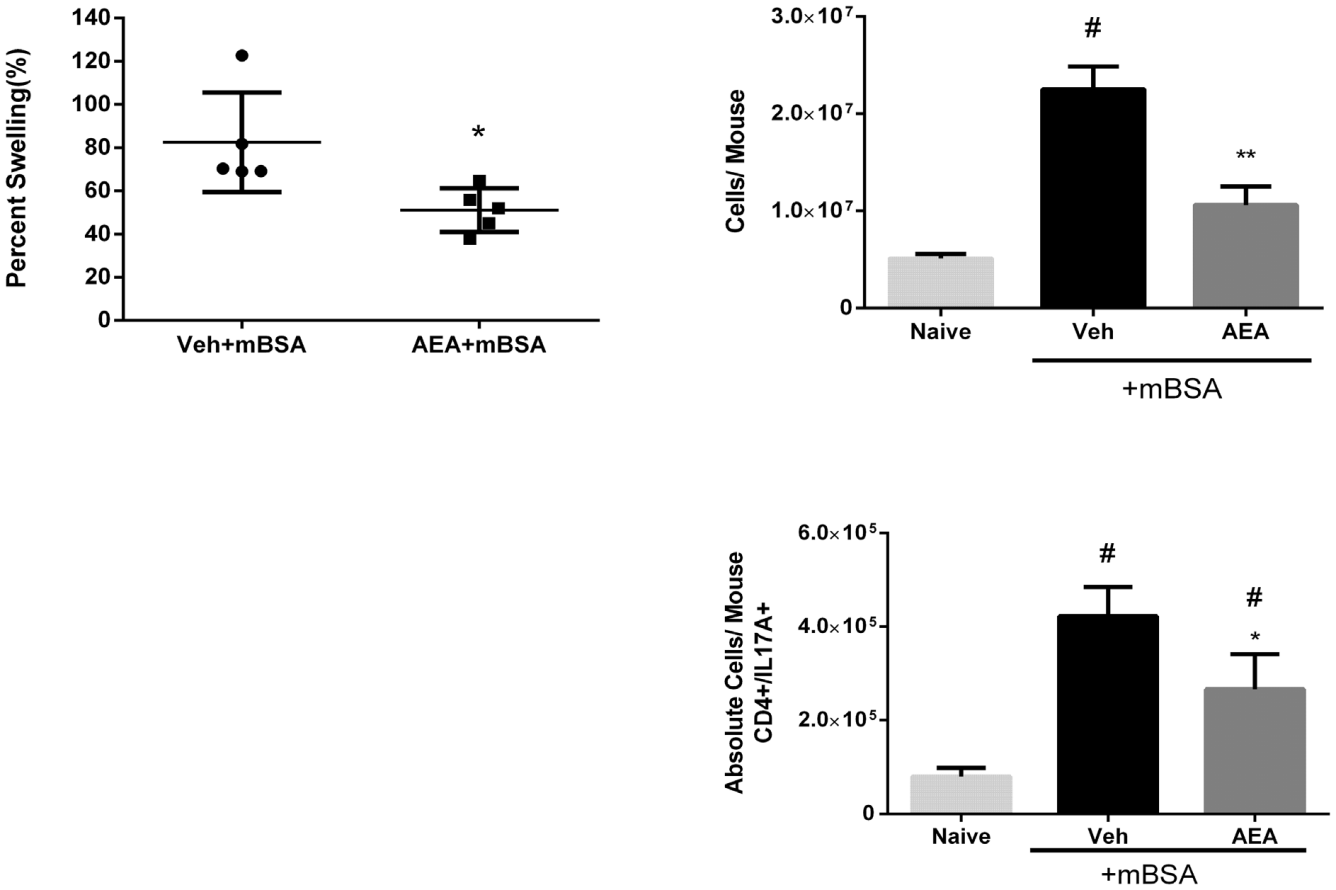
AEA ameliorates mBSA-Induced delayed hypersensitivity. Mice were sensitized to mBSA by giving an injection of 150 μg of mBSA emulsified into CFA into each hind flank. They were treated with AEA or vehicle on days 5 and 6 after sensitization and rechallenged with 200 μg mBSA into the right footpad and PBS in the left footpad. [A] The footpad swelling was measured as described in Methods. Graph representative of 3 independent experiments with n = 5. *p<0.05 compared to Veh. [B] Cells were harvested from draining lymph nodes [LN] and viable cells counted using trypan blue exclusion. Cell numbers are representative of 2 independent experiments with n = 5. #p<0.05 compared to naïve. **p<0.05 when compared to vehicle. [C] Cells from the LN were cultured for 4 hours in the presence of PMA, Ionomycin, and Golgi Plug and stained for IL-17 secreting cells. Graph is representative of 3 independent experiments with n = 3. # p<0.05 compared to naïve; *p<0.05 compared to vehicle.

**Figure 2:**
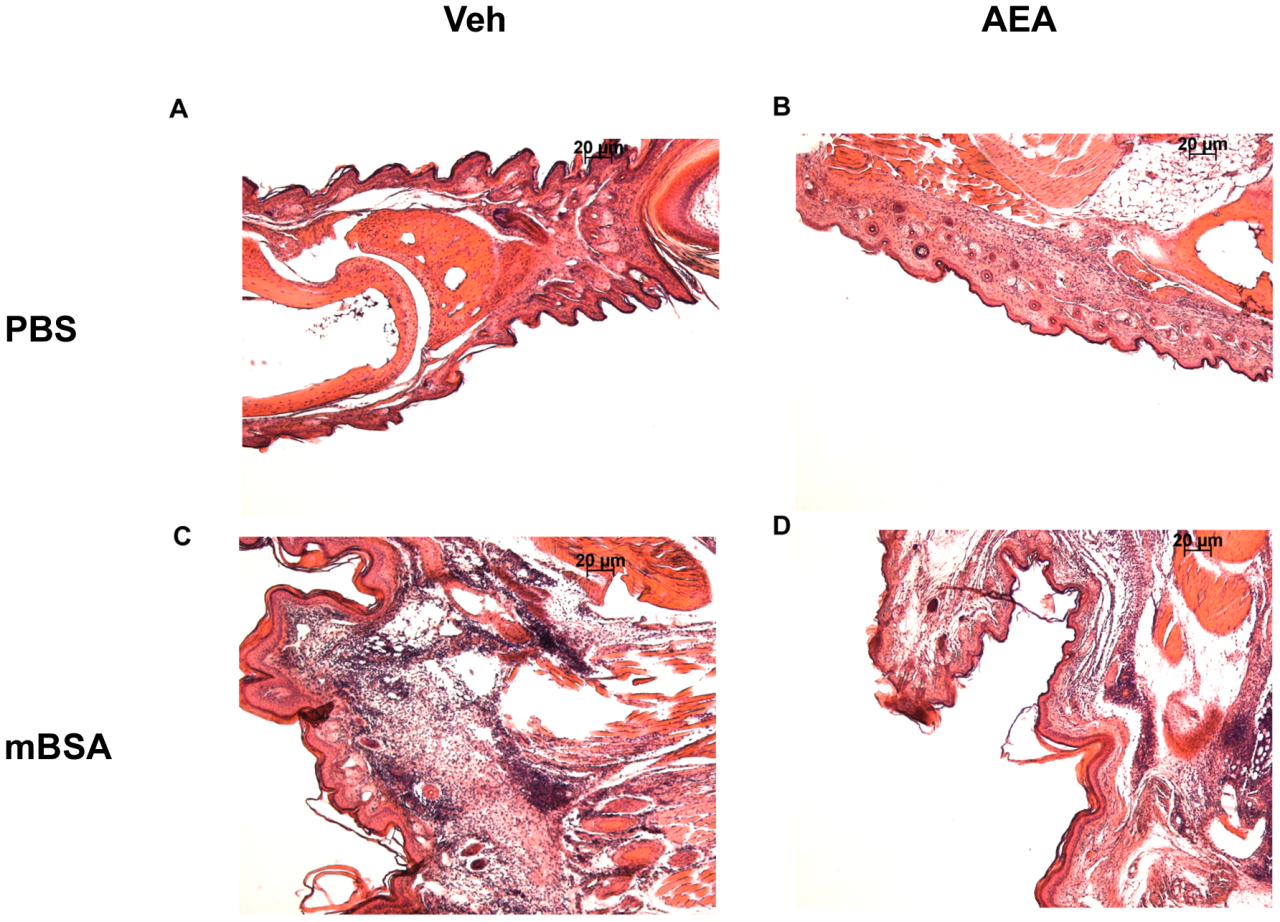
Histopathological analysis of mBSA rechallenged footpad. Mice were sensitized with mBSA and treated with either vehicle [A, C] or AEA [B, D], and rechallenged with either PBS [A, B] or mBSA [C, D], as described in Fig 1. The footpad was isolated and fixed/decalcified in Cal-Rite. The footpad was then embedded in paraffin and sectioned into 6μm sections and stained with Hematoxylin and Eosin. Pictures are representative of 2 individual experiments with n = 5. Magnification: 10×.

The endogenous cannabinoid system is important for regulating the hypersensitivity response [10], although the precise mechanisms remain unclear. In the current study, we tested the effect AEA, an endocannabinoid, on mBSA-induced Type-IV hypersensitivity in mice, which is mediated primarily by IL-17-producing T cells [11]. To this end, we sensitized mice to mBSA and treated them with AEA or vehicle for two days prior to rechallenge. Each mouse was rechallenged in the right footpad with mBSA and in the left footpad with vehicle, as described in Methods. We found that AEA-treated mice had significantly less swelling index in mBSA rechallenged footpads than vehicle-treated mice [Fig 1A], indicative of decreased inflammation. Furthermore, draining lymph nodes were taken and cells were counted to assess the lymph node cell proliferation in response to mBSA. Lymph node cells from AEA-treated mice showed decreased cell yield than vehicle-treated mice [Fig 1B], indicative of decreased response to mBSA. Next, we stained the lymph node cells for intracellular IL-17 by flow cytometry and calculated the absolute numbers of Th17 cells. mBSA immunization caused significant increase in IL-17 producing cells when compared to naïve mice, and AEA treatment significantly reduced the number of IL-17 producing cells [Fig 1C]. Histopathological analysis of footpads showed that mBSA treatment triggered strong infiltration of immune cells which was decreased following AEA treatment [Fig 2]. There was also less edema in AEA treated footpads. AEA effectively limited the disease manifestations of mBSA-induced hypersensitivity.

### AEA treatment skews away from proinflammatory cytokine production

**Figure 3:**
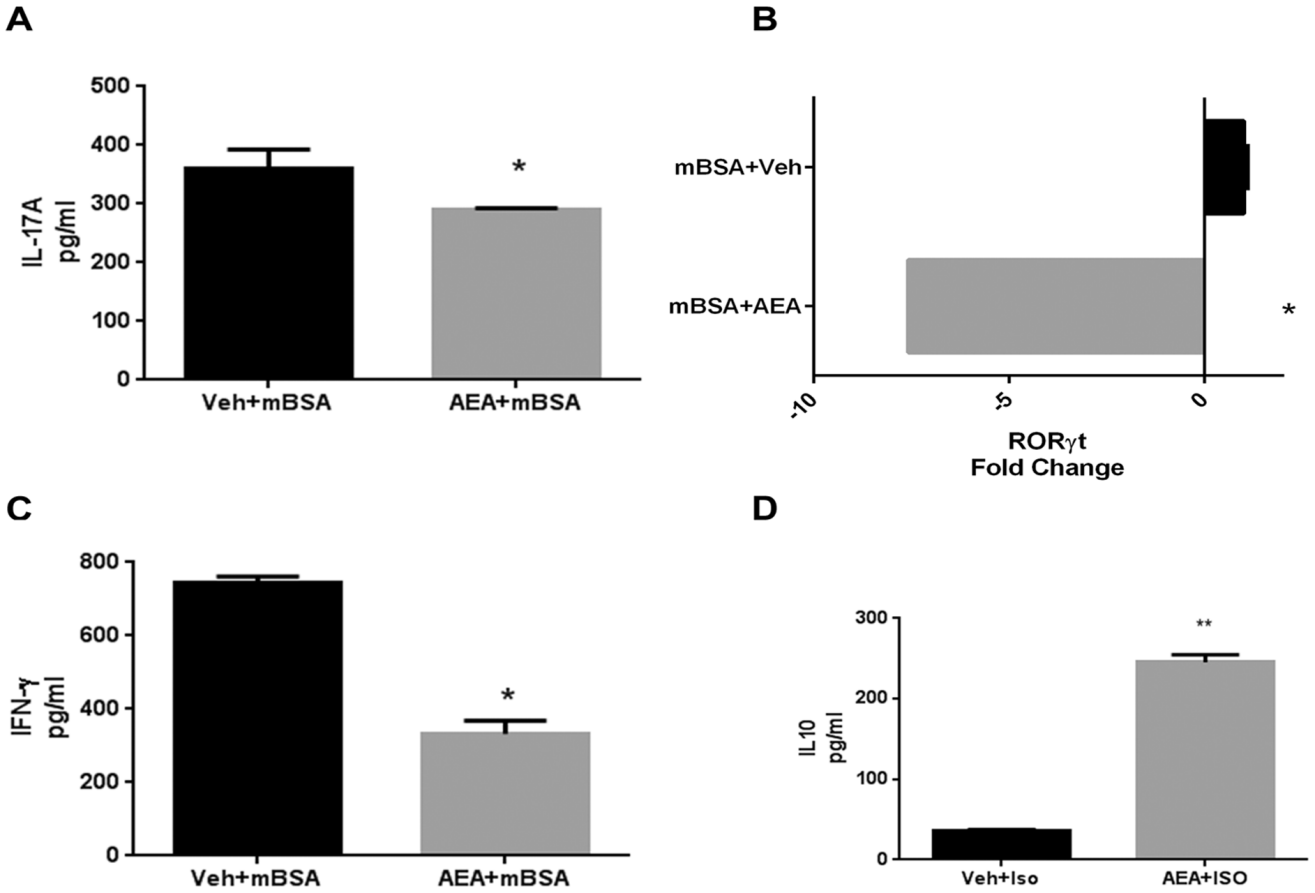
AEA treatment induces IL-10 expression, while suppressing IL-17 and IFN-γ. DTH response against mBSA was induced as described in Fig 1. Cells from draining lymph nodes of mBSA-rechallenged mice were isolated and cultured overnight to allow for spontaneous cytokine secretion. The culture supernatants were then analyzed for IL-17 [A], IFN-γ [C], and IL-10 [D]. ELISA data are representative of 3 individual experiments with n = 3. [B] RNA was isolated from LN of mice treated with AEA or Vehicle to analyze the expression of RORγt by RT-PCR. PCR plot is representative of 3 individual experiments with n = 3. *p<0.05 **p<0.01 when compared to vehicle.

We next tested for cytokine production, specifically both pro- and anti-inflammatory, from draining LN cells of mBSA-immunized mice treated with AEA or vehicle. To that end, we sensitized mice to mBSA and treated them with AEA or vehicle as before. We sacrificed these mice 24 hours after restimulation with mBSA and plated draining LN cells in complete RPMI overnight to allow for spontaneous cytokine secretion and then measured the levels of cytokines by ELISA. We found that AEA treatment significantly reduced IL-17 [Fig 3A] and IFN-γ [Fig 3C] secretion, as well as reduced RORγt expression [Fig 3B]. We also found that AEA treated cells produced significantly more IL-10 than vehicle controls [Fig 3D]. These data suggested that AEA decreases the induction of proinflammatory cytokines such as IL-17and IFN-γ and that this may result from increased IL-10 production.

### AEA mediates decrease in mBSA-Hypersensitivity by IL-10 secretion

**Figure 4:**
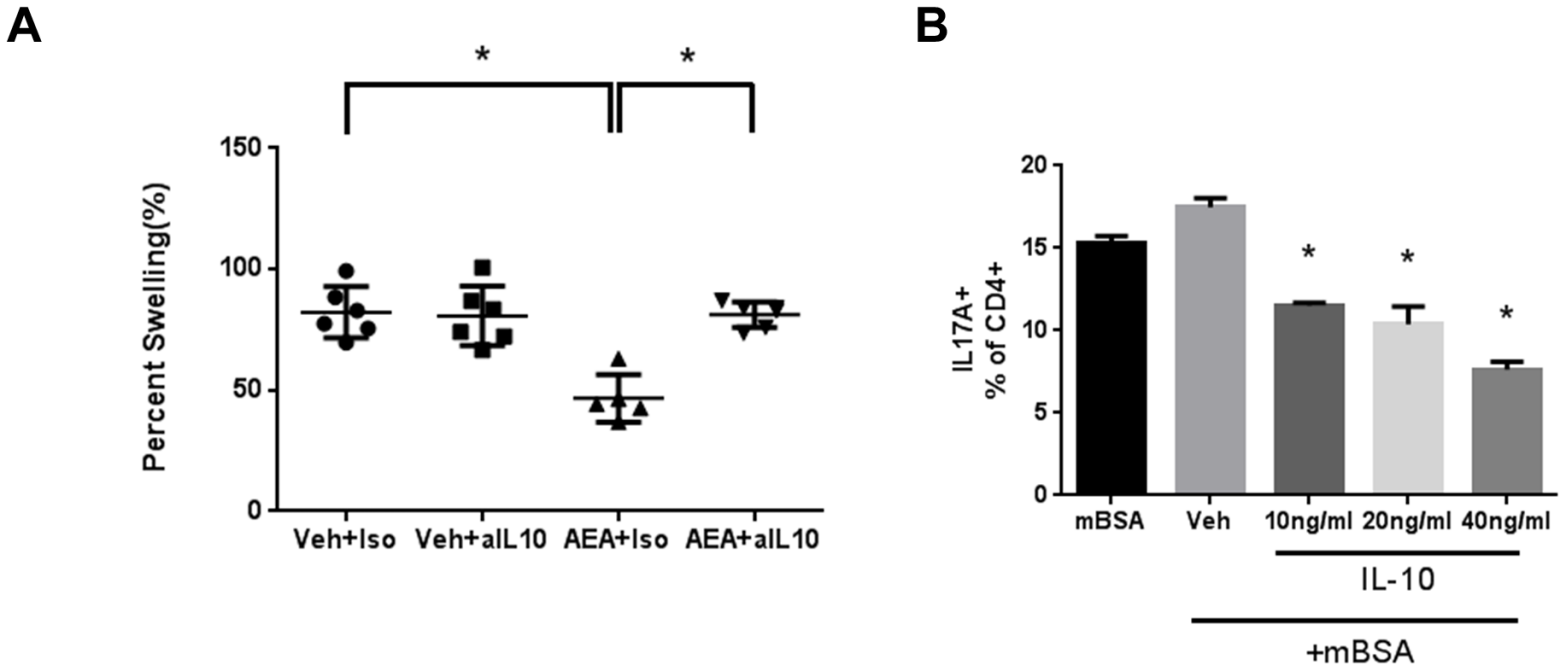
AEA suppresses DTH through IL-10 secretion. [A] Mice were sensitized to mBSA and treated with Vehicle or AEA, as described in Fig 1. An IL-10 neutralizing antibody or isotype control was given i.p. 1 hour before each treatment with AEA or vehicle, and [A] footpad swelling was measured. Footpad swelling data are a combination of 2 individual experiments with n = 3 for Veh and n = 3 for AEA. One mouse was excluded in both AEA treated groups because it was unclearly labeled. [B] Cells were isolated from the LN of mice sensitized to mBSA, plated, and cultured as described in Fig 3. rIL-10 was added to these cultures at 10, 20, or 40 ng/ml to assess the ability for IL-10 to knockdown the mBSA-induced Th17 response. Cells were cultured for 24 hours, and in the last 4 hours of culture, the cells were stimulated with PMA, Ionomycin, and Golgi Plug. Cells were harvested and stained for IL-17+ CD4 T cells. Data are representative of 3 individual experiments with n = 3. *p<0.05.

To directly test the role of IL-10, we sensitized mice to mBSA and treated them with AEA or vehicle as before. Next, we injected the mice with a neutralizing antibody to IL-10 [JES5-16E3] or isotype control antibody 1 hour after treatment with AEA or vehicle. We found that neutralizing IL-10 in vivo resulted in complete reversal of AEA-induced immunosuppression [Fig 4A]. Next, we cultured lymph node cells from mBSA-sensitized mice as before. We then treated those cells with increasing concentrations of rIL-10 to test the ability for IL-10 to suppress mBSA-induced IL-17 production. IL-10 was able to decrease IL-17-producing cells in a dose dependent fashion [Fig 4B].

### miRNA analysis of AEA treated mBSA mice

**Figure 5:**
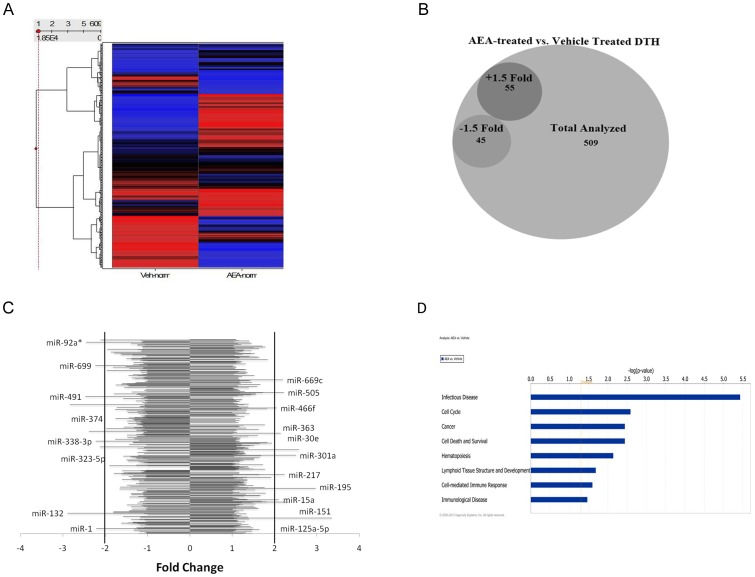
Effect of AEA on miRNA induction in mice with mBSA-induced DTH: Affymetrix miRNA array analysis. [A] Heat map of miRNA expression patterns. RNA was isolated from five pooled popliteal lymph nodes of mBSA-sensitized mice rechallenged with mBSA and treated with either AEA or Vehicle. The samples were analyzed using Affymetrix miRNA 1.0 microarray. The values for each sample were generated by normalizing the expression of the microRNA to each sample. Ward's Method was used for clustering patterns and the samples were ordered by the Input Rank method. [B] AEA induced differential regulation of several miRNA species, as enumerated by the Venn diagram. [C] The most differential regulated miRNA expressed as a bar chart. [D] Ingenuity Pathway Analysis of the most differentially regulated biological networks.

Recent studies have shown that microRNAs [miRNA] can regulate cytokines and inflammatory diseases [25]-[27]. To test if the immunosuppression by AEA was mediated by miRNA, we isolated total RNA from draining lymph nodes in mice sensitized to mBSA and treated with vehicle or AEA, as before. We analyzed those samples using Affymetrix miRNA 1.0 microarray. We analyzed 609 different miRNA species for changes in AEA treated mice compared to vehicle controls [Fig 5A]. We found that AEA showed a different expression pattern of miRNA species than vehicle-treated mice. Specifically, there were 100 miRNA species that were differentially regulated in AEA-treated animals, 55 upregulated 1.5 fold or greater and 45 downregulated 1.5 fold or greater [Fig 5B]. We next looked for the most differentially regulated miRNA species. We found that AEA highly upregulated the expression of miRNA 125a-5p [+3.35], miRNA 301a [+2.50], miRNA 30e [+2.57], miRNA 151 [+2.32], and many others. Conversely, AEA downregulated miRNA 374 [−2.38], miRNA 491 [−2.46], and miRNA 132 [−2.89], among others [Fig 5C]. Using Ingenuity Pathway Analysis, we found that AEA significantly affected several miRNA-regulated canonical biological pathways [Fig 5D]. Among these, Infection Disease, Cell Cycle, Cell Death and Survival, and the Cell-mediated Immune Response were of interest in our system.

### Role of IL-10-induced miRNA in the regulation of inflammation

**Figure 6:**
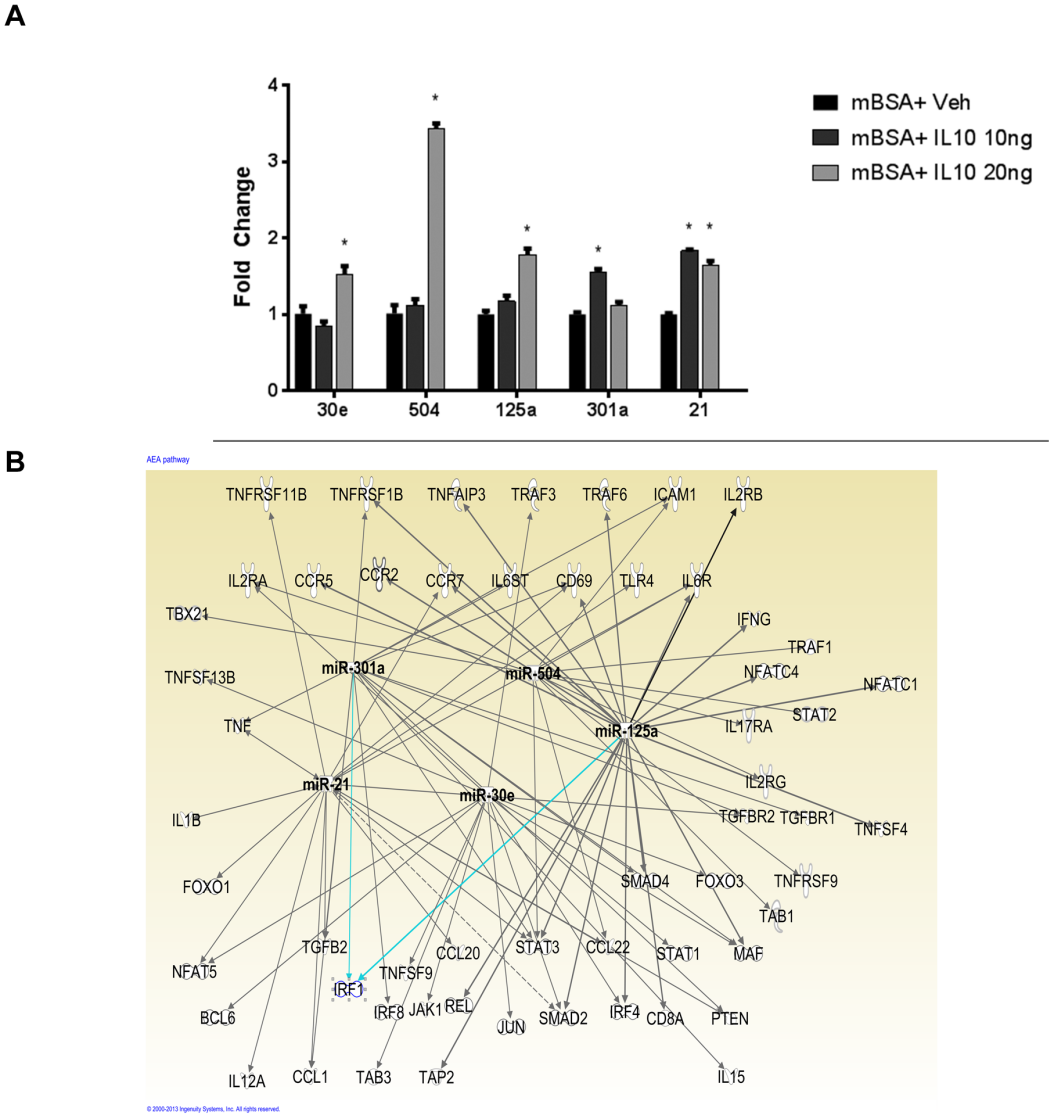
IL-10 induces miRNA that affect proinflammatory mediators. [A] RNA was isolated from mBSA rechallenged LN cells treated with vehicle or IL-10 [20, 10 ng/ml], as described in Fig 4. qPCR was performed to analyze changes in miRNA 21, 504, 125a, 30e, and 301a. Data are representative of 3 individual experiments with n = 3. [B] Ingenuity pathway analysis [IPA] of miRNA targets for miRNA induced by IL-10. * p<0.05 compared to vehicle.

Because we noted that IL-10 plays a crucial role in the regulation of anti-inflammatory effects of AEA, we next determined if IL-10 treatment induces miRNA that can suppress inflammatory pathways. To this end, we isolated RNA from mBSA sensitized cells treated with vehicle or rIL-10 at 10 and 20 ng/ml and analyzed them for specific miRNA that we identified from our in vivo data which could potentially target inflammatory pathways [Fig 5C]. Interestingly, we found that IL-10 was able to induce the expression of miRNA 30e, 504, 125a, 301a, and 21 [Fig 6A]. It was noteworthy that several of these miRNAs such as 30e, 125a, and 301a had been found to be up-regulated in vivo upon AEA treatment [Fig 4C]. We then used Ingenuity pathway analysis to determine the effect of these miRNAs on pro-inflammatory mediators [Fig 6B]. Interestingly, these microRNAs were found to target several proinflammatory cytokines and their receptors, including IFN-γ, TNF-α, and TGF-β/IL-6 necessary for Th17 differentiation. Along with these, STAT-3 was also targeted by several of these miRNAs. Together, these data suggested that AEA may promote IL-10 induction which in turn may up-regulate certain miRNA that suppress inflammatory pathways.

## Discussion

Our current study shows that when endogenous cannabinoids are given as a treatment they can attenuate Th17 cell-mediated inflammation and the subsequent mBSA-induced hypersensitivity. AEA treatment effectively limited measurable inflammatory parameters, including footpad swelling, draining LN cell proliferation, and histopathological cell infiltration and tissue injury. We found that treatment of AEA in vivo decreased the generation of proinflammatory IFN-γ and IL-17 producing cells, while increasing IL-10 production. The induction of IL-10 was found to be critical for AEA-induced attenuation of the hypersensitivity response inasmuch as blocking IL-10 reversed the suppression of hypersensitivity caused by AEA. Finally, we showed that IL-10 directly induced several different microRNAs that targeted proinflammatory cytokines. Interestingly, miRNA analysis of cells from mice treated with AEA showed significant alterations in several miRNAs, some of which correlated with in vitro induction following IL-10 exposure. These data together suggested AEA suppresses inflammation through induction of IL-10, which mediates its effect by triggering several microRNAs that target proinflammatory cytokine pathways. Our data suggest that miRNA regulation serves as a potential mechanistic link between IL-10 and reduction of IL-17 and other proinflammatory cytokines such as IFN-γ. This study opens several avenues toward understanding the role of the endocannabinoid system and miRNA in regulating the immune response. While we studied the proinflammatory cytokine-induced delayed hypersensitivity response in this study, it is not clear if endocannabinoids would affect other inflammatory markers such as Serum Amyloid A or C -Reactive Protein, markers for systemic inflammation, and additional studies are necessary to address this.

Studies from our laboratory and elsewhere have characterized cannabinoids as potent immunosuppressive agents [30]–[35]. Δ-9-tetrahydrocannabinol has been shown to induce apoptosis in dendritic cells via NF-kB [30], induce apoptosis in splenocytes [31], and mediate a cytokine shift from Th1 to Th2 cytokines [32]. Cannabinoids also induce populations of immunosuppressive cells including myeloid derived suppressor cells [33] and regulatory T cells [34], [35]. While the mechanism for exogenous cannabinoid-mediated immune suppression is well characterized, remarkably little has been reported about the mechanisms involved in the regulation of immune system by endocannabinoids.

Mice lacking both known cannabinoid receptors were shown to display enhanced cutaneous contact hypersensitivity [10]. In the same study, mice with increased levels of the AEA displayed reduced allergic responses in the skin. These results demonstrated a protective role of the endocannabinoids in contact hypersensitivity. Endogenous cannabinoids have been implicated as a putative therapeutic modality in immunological disease [36], [37]. AEA has been reported to induce apoptosis and inhibit proliferation in lymphocytes [38]. Furthermore, anandamide affects the secretion of Th1 cytokines in peripheral mononuclear cells [39]. Fatty Acid Amide Hydrolase [FAAH] is also down regulated in response to LPS in human lymphocytes, leading to an increase in AEA [40]. Activation of the endocannabinoid system modulates macrophage activity in coronary artery disease [41], as well as affecting dendritic cells [42]. However, to the best of our knowledge, no mechanism has been described that linked the endogenous cannabinoid system to regulation of Th17 driven inflammation.

IL-10 was first described as an inhibitory factor secreted by Th2 cells that inhibited the secretion of cytokines by Th1 cells [43]. Cannabinoids have been linked to increased expression of IL-10 [44], [45]. Recently, IL-10 signaling has been shown to be important for inhibition of Th17 mediate inflammation as well [46]. However, the precise mechanisms of IL-10-mediated inhibition of Th1 and Th17 inflammation are not well understood. It is known that IL-10 induces Gata-3 transcription [47]. Gata-3 is well known to inhibit the differentiation of other T helper cell lineages [48]. Using Theiler virus model of MS, it was shown that AEA enhanced IL-10 production by microglia and suppressed IL-12p70 and IL-23 [49]. In the current study, we noted that neutralization of IL-10 in vivo, reversed the immunosuppressive effects of AEA, thereby suggesting the critical role played by IL-10. Moreover, our studies showed that IL-10 may regulate its effect through induction of miRNA species.

microRNAs play a critical role in T helper cell differentiation [25]–[27]. Our studies show that IL-10 induces specific microRNA, including miRNA 21, 504, 301a, 30e, and 125a that target genes important for the induction of other T helper subsets. miRNA 21 has been linked to inflammation through a variety of mechanism. Upregulation of miRNA 21 targets IL-12 in anti-mycobacterial responses, leading to impaired T cell anti-mycobacterial responses [50]. miRNA 21 is also found to target TNF-α, IL-1β, TGF-β, and IL-6R according to Ingenuity pathway analysis. IL-1β has been shown to be critical for the induction of a delayed-type hypersensitivity response to mBSA [51]. Furthermore, IL-1β has been shown to promote the secretion of IL-17a by the induction of IRF4 [52], [53]. miRNA 504 is a relatively unknown species of microRNA, with no previous reports about its role in inflammation. Our IPA analysis suggested that miRNA 504 targets several important molecules, including T-bet and STAT-3. T-bet is the master regulator for Th1 differentiation [54], and drives the immune response toward a proinflammatory environment. This could be a potential mechanism through which IL-10 inhibits differentiation of Th1 lineage, by direct binding and inhibiting T-bet.

The Ingenuity pathway analysis showed a possible effect of AEA on cell cycle progression. This effect has been noted previously, as cannabinoids have been reported to affect the cell cycle in many different cell types. Cannabidiol [CBD] inhibits proliferation of MOG-specific T cells in a mouse model of MS [55]. Endocannabinoids have also been shown to have this ability; 2-AG inhibits the activation and proliferation of U87MG glioma cells in response to peptidoglycan [56]. AEA inhibits the proliferation of human prostate cancer cell lines by downregulating epidermal growth factor receptor [57]. These studies corroborate the role of cannabinoids in cell cycle/proliferation pathways as suggested by pathway analysis.

In summary, our studies show that treatment with endocannabinoids leads to an increased secretion of IL-10, which triggers miRNA that negatively target IL-17 and other proinflammatory pathways, thereby suppressing delayed type hypersensitivity response. These studies open the door for further examination of these miRNAs and their target genes to identify new therapeutic targets to treat Th17-driven autoimmunity, such as Multiple Sclerosis. These studies also suggest that endogenous cannabinoid system is one of the homeostatic mechanisms that the body employs to down-regulate immune response to foreign antigens as well as combat autoimmunity. Targeting of this system could yield valuable therapeutics in the future.
